# Antibody-Based HIV-1 Vaccines: Recent Developments and Future Directions: A summary report from a Global HIV Vaccine Enterprise Working Group

**DOI:** 10.1371/journal.pmed.0040348

**Published:** 2007-12-01

**Authors:** David Montefiori, Quentin Sattentau, Jorge Flores, José Esparza, John Mascola, on behalf of a Working Group convened by the Global HIV Vaccine Enterprise

## Abstract

The authors discuss humoral immune responses to HIV and approaches to designing vaccines that induce viral neutralizing and other potentially protective antibodies.

The Global HIV Vaccine Enterprise convened a two-day workshop in May of 2007 to discuss humoral immune responses to HIV and approaches to design vaccines that induce viral neutralizing and other potentially protective antibody responses. The goals of this workshop were to identify key scientific issues, gaps, and opportunities that have emerged since the Enterprise Strategic Plan was first published in 2005 [1], and to make recommendations that Enterprise stakeholders can use to plan new activities.

Most effective viral vaccines work, at least in part, by generating antibodies that inactivate or neutralize the invading virus, and the existing data strongly suggest that an optimally effective HIV-1 vaccine should elicit potent antiviral neutralizing antibodies. However, unlike acute viral pathogens, HIV-1 chronically replicates in the host and evades the antibody response. This immune evasion, along with the large genetic variation among HIV-1 strains worldwide, has posed major obstacles to vaccine development. Current HIV vaccine candidates do not elicit neutralizing antibodies against most circulating virus strains, and thus the induction of a protective antibody response remains a major priority for HIV-1 vaccine development. For an antibody-based HIV-1 vaccine, progress in vaccine design is generally gauged by in vitro assays that measure the ability of vaccine-induced antibodies to neutralize a broad spectrum of viral isolates representing the major genetic subtypes (clades) of HIV-1 [2]. Although it is not known what magnitude and breadth of neutralization will predict protection in vaccine recipients, it is clear that current vaccine immunogens elicit antibodies that neutralize only a minority of circulating isolates. Thus, much progress needs to be made in this area. Also, though virus neutralization is considered a critical benchmark for a vaccine, this may not be the only benchmark for predicting success with antibody-based HIV-1 vaccine immunogens.

The main targets for neutralizing antibodies to HIV-1 are the surface gp120 and trans-membrane gp41 envelope glycoproteins (Env) that mediate receptor and coreceptor binding and the subsequent membrane fusion events that allow the virus to gain entry into cells [3]. Antibodies neutralize the virus by binding these viral spikes and blocking virus entry into susceptible cells, such as CD4^+^ T cells [4,5]. In order to chronically replicate in the host, the virus exploits several mechanisms to shield itself against antibody recognition, including a dense outer coating of sugar molecules (N-linked glycans) and the strategic positioning of cysteine–cysteine loop structures on the gp120 molecule [6–8]. These shielding mechanisms, although highly effective, have vulnerabilities imposed by fitness constraints. Information on the precise location and molecular structure of these vulnerable regions could be valuable for the rational design of improved vaccine immunogens.

Participants in the workshop identified four areas that, if given proper attention, could provide key information that would bring the field closer to an effective antibody-based HIV-1 vaccine: (1) structure-assisted immunogen design, (2) role of Fc receptors and complement, (3) assay standardization and validation, and (4) immunoregulation of B cell responses.

## Structure-Assisted Immunogen Design

Clinical studies have demonstrated that immunization with the gp120 surface unit of the HIV-1 envelope protein does not lead to the induction of potent or broadly reactive neutralizing antibodies. In order to develop better immunogens, it is likely that we will need a more detailed understanding of the atomic level structure of epitopes on the native envelope glycoprotein. Data on the X-ray crystal structure of liganded and unliganded partial gp120 molecules have provided valuable information about the atomic level interaction of gp120 and neutralizing antibodies [9–12]. The recent atomic level resolution of monoclonal antibody (MAb) b12 bound to the CD4 receptor binding site of the gp120 molecule provides new insights into how successful neutralizing antibodies access functionally conserved regions of the Env glycoprotein [13]. Crystal structures of complete monomeric gp120 and gp120–gp41 trimer complexes in their native unliganded form need to be elucidated, as these are the natural targets for neutralizing antibodies. This information is needed for multiple genetic subtypes of the virus and for transmitted strains of the virus. Coupled with this effort should be a program to make necessary improvements in electron tomography technology to gain a higher resolution of native Env spikes as they exist on virus particles [14–16]. An improved understanding of the structural basis of antibody binding to the HIV-1 Env glycoprotein will likely form the foundation for a rational program of novel vaccine design. Ongoing efforts to stabilize gp120 into more immunogenic forms or to scaffold conserved neutralization epitopes into foreign proteins may lead to more promising antibody responses.

Induction of an effective neutralizing antibody response will require that a vaccine deliver to the naïve B cell repertoire epitopes that are both immunogenic (i.e., possess favorable properties for B cell inductive pathways) and antigenic (i.e., available for high affinity antibody binding on functional Env spikes). Viral epitopes that are conserved among most viral strains are more likely to generate cross-reactive antibodies. In this regard, researchers have focused on a small number of human MAbs, from clade B HIV-1-infected individuals, that possess broadly cross-reactive neutralizing activity [17,18]. The cognate viral epitopes for these MAbs have been well characterized and are being evaluated as vaccine immunogens. However, for reasons that are not completely understood, these conserved viral epitopes have either been poorly immunogenic or have elicited antibodies of restricted reactivity. Improvements are being sought by introducing specific structural alterations [19,20] and by targeting autoreactive B cell pathways [21]. These and other efforts to improve the immunogenicity of conserved neutralization epitopes should remain a high priority. Workshop participants recognized the need to expand efforts to identify and characterize new MAbs, with special attention to MAbs from non-clade B HIV-1 infections. New technologies are now available that might afford an advantage for identifying novel antibody specificities that were previously undetected [22,23]. In addition to this focus on MAbs, sera from selected HIV-1-infected individuals that can broadly neutralize HIV-1 isolates should be studied in detail. New assays allow more precise mapping of the polyclonal antibody response in these sera to better understand the epitopes targeted [5,24–26]. Such studies may reveal novel antibody specificities and their associated viral epitopes that could be useful for immunogen design.

While there has been considerable interest in conserved epitopes, less attention has been paid to more variable epitopes that might be useful if administered in the form of a polyvalent vaccine. Of particular interest are the epitopes that drive the autologous neutralizing antibody response in infected individuals. These epitopes may be quite variable, but recent evidence suggests that there are constraints on the extent of variation the virus can tolerate in these regions [27,28]. Detailed molecular and immunologic studies of the autologous neutralization response would enhance our understanding of viral determinants that are vulnerable to antibody attack. Similarly, it is possible that combinations of antibodies will have desirable additive or synergistic effects on virus neutralization [29–32]. An example is seen in how soluble CD4 binding rearranges the structure of gp120 to expose the highly conserved coreceptor binding domain, which allows antibody binding and virus neutralization to occur [33,34]. Such effects of antibodies might be discovered by applying high throughput screening methods to the plethora of existing MAbs as well as new MAbs that become available in the future.

## Role of Fc Receptors and Complement

Recent findings have generated renewed interest in so-called “non-neutralizing” antibodies that are unable to directly inhibit free virus entry into target cells, but nonetheless exhibit antiviral activity mediated by the Fc region of the antibody molecule. These antibody effector mechanisms include complement binding and viral lysis, phagocytosis of antibody-coated virions, and antibody-dependent cellular cytotoxicity [35–38]. Recent studies have suggested examples of Fc-dependent antiviral effects of HIV-1-positive serum in cases where there was little or no detectable activity in conventional neutralization assays [39,40].

In addition, passive transfer studies in a relevant monkey model suggest that Fc receptor (FcR) binding capacity of a protective antibody makes a substantial contribution to the antibody-mediated protection [41]. Antibody effector functions that mediate complement activation and FcR engagement on macrophages, dendritic cells, natural killer cells, and other cell types need to be evaluated to determine their relevance to HIV-1 vaccines. Assays that measure these antiviral antibodies should be standardized and used to assess biologic relevance in passive protection experiments in animal models using antibodies that exhibit the different effector functions in vitro.

## Assay Standardization and Validation

In order to adequately monitor neutralization breadth and potency and to compare and prioritize immunogens, assays are needed that are sensitive, quantitative, high throughput, and have correlative value. Substantial improvements have been made in the past several years in assay technology and in available reference reagents. Thus, cumbersome and expensive assays using peripheral blood mononuclear cells (PBMC) and uncloned viruses are being replaced with assays that utilize molecularly cloned Envpseudotyped viruses and genetically engineered target cells lines [2,42–45]. This new technology affords greater sensitivity, reproducibility, high throughput, and cost-effectiveness compared to PBMC assays, and as a result, it has been responsible for an explosion of new data. Steps are being taken by the Collaboration for AIDS Vaccine Discovery to transfer this new technology to multiple laboratories around the world and to implement a validated proficiency testing program to assure inter-laboratory equivalency in assay performance.

Recently, several cases were identified where neutralization was considerably more potent or only detected in the older PBMC assay compared to the newer assay technology [43,46,47]. This raises important questions about current plans to employ a single assay for routine use, and it points to the need for a better understanding of the mechanisms of neutralization. Thus, it may be necessary to use more than one assay to assure that all neutralizing antibodies are detected. There is a need to standardize and compare neutralizing antibody assays and to decide which assay or combination of assays should be used for standardized assessments of vaccine-elicited neutralizing antibody responses. A major priority is to strengthen the standardization of the PBMC assay, given that it is the only assay that has been at least partially validated in passive antibody experiments in animal challenge models.

Important decisions need to be made about the types of antibodies and assays that have greatest relevance to HIV-1 vaccines. Validation experiments in animals models are needed to determine the potential correlative value of new assay technologies that rely on the use of genetically engineered cells lines and Envpseudotyped viruses. Ideally, this would be done by employing several different assays to study the antibody response in a clinical efficacy trial in which the vaccine was at least partially protective. Because no such vaccine is currently available for HIV-1, studies in animal models are the next best choice. In this regard, two animal models are widely used for HIV vaccine development: simian immunodeficiency virus (SIV) and chimeric simian-human immunodeficiency virus (SHIV) infection in monkeys [48]. Quantitative passive transfer experiments in either model with antibodies that exhibit different effector functions could be used to address the biological relevance of in vitro assays. Unfortunately, very few SHIVs are currently available and, among these, most are derived from a single genetic subtype (clade B) and exhibit properties that may not be well suited to assay validation [49]. The creation of new and better SHIVs from non-clade B viruses would facilitate assay standardization as well as vaccine challenge models.

## Immunoregulation of B Cell Responses

This workshop identified several critical gaps in the current understanding of B cell regulatory pathways that impede a more rational development of an effective antibody-based HIV-1 vaccine. For example, broadly neutralizing antibodies in patient serum bind epitopes that are present on monomeric gp120 [25], yet this is a poor immunogen for neutralizing antibody induction in vaccine recipients. Moreover, as mentioned above, viral epitopes for the known broadly neutralizing MAbs appear to be poorly immunogenic in infected individuals and as vaccine candidates. Insights into the immunoregulation of some of these latter epitopes (e.g., epitopes defined by MAbs 2F5 and 4E10) was provided by recent studies in which the MAbs were discovered to bind one or more self antigens [50,51], raising the possibility that these antibody specificities are subjected to negative regulation mechanisms, such as receptor editing or deletion. Thus, Env as an immunogen may bypass key steps in the B cell inductive pathway, or may actively induce negative production or downregulation of production of some broadly neutralizing antibodies [52–54].

The receptor–ligand interactions and intracellular signaling pathways that govern the production of antibody-producing plasma cells and the persistence of plasma and memory B cells are poorly understood. Additional information on the mechanisms responsible for B cell migration, selection, and differentiation within and between specialized anatomical sites, particularly within lymphoid follicles, might be used to target suitable Env epitopes to appropriate B cell inductive pathways. An example would be to provide necessary signals to generate long-lived and high affinity memory in the marginal zone B cell compartment. Another example would be to discover ways to modify germinal center formation, positive and negative selection, and B cell differentiation to drive long-lived high affinity antibody responses against key epitopes that tend to be poorly immunogenic.

In parallel to these efforts, genetic studies at the population level could provide critical information on the most promising paths to follow. In particular, the recent completion of the International HapMap Project now permits whole genome associated studies to be conducted with a minimum number of single nucleotide polymorphism tags [55,56]. This powerful new technology could be used to identify genes that are associated with the wide variation in neutralizing antibody responses in HIV-1-infected individuals and in vaccine recipients. A critical question to ask is whether the potent neutralizing antibody response in a small subset of infected individuals is due to unique viral epitopes or to host genetic polymorphisms. Current evidence suggests that both might make a substantial contribution in the context of combined epitope and allelic representations [28,47,57].

To date most studies of the humoral responses in HIV infections have investigated immunoglobulins, the final product of B cell responses. Relatively few studies have examined B cell immunopathogenesis. A number of basic questions are still unanswered (e.g., extent and reason for perturbation of B cell subset changes, including memory B cells and plasma cells, in peripheral blood and tissues). Questions also remain about other potential functional contributions of B cells to HIV infections (e.g., role as antigen-presenting cells). In vivo studies should be performed in the nonhuman primate animal model to determine the emergence of pathologic events in the B cell compartment, in particular in lymphatic and gastrointestinal tissues of naïve and vaccinated animals that are challenged with pathogenic SIV or SHIV. These investigations should be done in parallel to detailed analyses of the magnitude and function of HIV-specific immunoglobulin responses determined in plasma and tissue secretions, and of HIV-specific B cells on a single cell basis.

The establishment of a research consortium to study fundamental B cell biology as it relates to HIV-1 vaccines is recommended. This program should be structured in a way that asks key scientific questions about B cell regulatory pathways that modulate Env immunogenicity. Studies could address B cell receptor–ligand interactions and intracellular signaling pathways that govern the production of antibody-producing plasma cells, the persistence of plasma and memory B cells, the mechanism of action of adjuvants, and host genetic associations with immune responses.

## Abbreviations

FcR: Fc receptor
MAb: monoclonal antibody
PBMC: peripheral blood mononuclear cells
SHIV: chimeric simian-human immunodeficiency virus
SIV: simian immunodeficiency virus
